# Health Care Violence and Abuse towards Nurses in Hospitals in North of Iran

**DOI:** 10.5539/gjhs.v5n4p211

**Published:** 2013-05-20

**Authors:** Mohammad Khademloo, Fatemeh Sheikh Moonesi, Hamed Gholizade

**Affiliations:** 1 Department of Community Medicine, Mazandaran University of Medical Sciences, Sari, Iran; 2 Department of Psychiatry, Psychiatry and behavioral Sciences Research Center, Mazandaran University of Medical Sciences, Sari, Iran

**Keywords:** violence, verbal, physical

## Abstract

**Aim:**

In the current survey, we explored the prevalence of verbal and physical abuse against the nurses in different hospitals of north of Iran.

**Methods:**

We performed a cross-sectional survey. Nurses were interviewed using a standardized questionnaire (Staff Observation Scale Revised (SOAS-R)). The sample covered 400 participants from 5 hospitals of Mazandaran University of medical sciences, Sari, Iran.

**Results:**

The sampling size involved 271 participants (271 forms from 400 forms (sampling population) were filled completely) including 193 female (71.2%) and 78 male (28.8%) participants. 79 (29.1%) participants experienced physical abuse and 260 (95.9%) participants were abused verbally. It was noted that in 35 cases (44.3%) the patients were the source of physical abuse and in 44 cases (55.6%) the members of patients’ family were the source. In 79 (30.3%) cases the patients were the source of verbal violence and in 139 (53.4%) cases the members of patients’ family were the source and in 42 (16.1%) cases coworkers were the sources.

**Conclusion:**

Verbal abuse was a common type of violence in our study in north of Iran. There is a requirement to increase awareness about this significant problem among health care workers.

## 1. Introduction

In recent years, job associated-violence was a complex and dangerous problem for the staffs (Beech et al., 2006; Cooper et al., 2002). Among different works, health care staffs were defined as one of the most groups that experience aggression (Bourn, 2003; Chappell et al., 2006; Wells et al., 2002; Çelik et al., 2012; Alencar et al., 2012).

Based on Nova Scotia Association of Health Organizations, violence is defined as any behavior which cause damage whether real or perceived. Researches revealed that the most frequent origin of abuse were patients, patients’ family, visitors, doctors, other healthcare staffs and even nurse co-workers (May et al., 2002; Oweis et al., 2005; Uzun et al., 2003).

Among the hospitals workers nurses confront with verbal, emotional, physical and sexual abuse (Uzun et al., 2003; Gerberich et al., 2005). Due to Their presence in stressful conditions including accidents, deaths, staying to visit a doctor, or transfer of patients to a ward predispose the nurses to more abuse from patients or their companions than other hospital staffs (Islam, 2003). The impact of abuse on nurses result in tiredness, sleeping disorders, nightmares, stress, continuous headaches, chronic aches, spasm, diminished self esteem, self dissatisfaction, disappointment, short-temperedness, symptoms of amnesia (after being hit), phobia, depression, alcohol intake, smoking, and sometimes suicide. Physical violence will cause physical disorders including backache, or even the death of a nurse (Anderson, 2002; Gates et al., 1999; Lee et al., 1999; Nolan et al., 2001; Pejic, 2005; Rippon et al., 2000).

There are different reports about the prevalence of abuse rate against nurses from 37% to 72% (Lin et al., 2005; Stirling et al., 2001).

Therefore in this study, we investigate the prevalence of verbal and physical abuse against the nurses in different hospitals of north of Iran which were related to Mazandaran University of Medical Sciences, Sari, Iran.

## 2. Methods and Material

### 2.1 Subjects and Data Collection

In this investigation, 400 nurses who were members of the Mazandaran University of Medical Sciences, sari, Iran took part as participants.

Questionnaires, along with approval forms, were sent to 400 nurses who were members of the university in various hospitals in charge of the Nursing Association. Nurses were informed that they could give back the forms to the head of the Nursing Association in related hospitals.

Exclusion criteria included who were mentally ill, and nurses with less than 1 year of job experience.

### 2.2 Participants Recruitment

The study included permanent staff who working continuously in the same post for at least one year. The questionnaires were distributed in informative sessions performed with groups of between 20 and 30 participants, who were informed with information about the research and details of how to response and hand in the study.

### 2.3 Survey Data (Questionnaire)

The experience with violence and aggressive behavior and the interactions performed for controlling such conditions were recorded by Staff Observation Scale Revised (SOAS-R) by Nijman and colleagues (Nijman et al., 1999) which was developed by researchers.

Characteristics of this questionnaire are its comprehensibility, and the psychometric feature. The questionnaire consists of 20 questions from three blocks of topics. The first block includes questions about the participant and their profession, like gender and age, the health care setting in which they work, their qualifications and experience at job.

In the second section, staff experience with violence and aggressive conditions is being evaluated. First participants are questioned separately about physically and verbally aggressive behavior: “Have you experienced physically aggressive behavior in the last twelve months?” and “Have you experienced verbal aggression in the last twelve months?”. More questions refer to the kind and purpose of the aggressive behavior. The last part of questionnaire includes the need for procedures to manage violence and aggression. This part contains questions about physical and emotional consequences, general stress associated with the incidents, help which is provided in the hospital and their utilization and social support.

### 2.4 Statistical Analysis

Data was collected, reviewed, coded and entered into the computer program. Data was presented in the form of frequencies and percentages. Chi-squared test was used for comparing qualitative data. Statistical analysis was done using the SPSS program version 16 (SPSS Ver 16).

### 2.5 Ethical Issues

All personal data of the participants were secret and no one has access to them except researchers.

## 3. Results

While 354 questionnaires were received, just 271 could be analyzed and interpreted because the rest were not filled in completely. The study population involved 271 participants with mean ± SD age of 36.8 ± 9.35 including 193 female (71.2%) subjects with mean ± SD age of 33.4 ± 7.85 and 78 male (28.8 %) participants with mean ± SD age of 38.36 ± 11.78. More than two-thirds (76%) were married at the time of survey, and 17.3 percent were single and the remaining 6.7% were separated/widow/divorced. The workplace of these people included emergency department 33 cases (12.2%), internal department 44 cases (16.2%), surgery department 46 cases (16.9%), gynecology department 10 cases (3.7%), psychology department 24 cases (8.9%), operating room 32 cases (11.8%), pediatrics department 17 cases (16.3%) and other department 65 cases (24%) (Table 1).

**Table 1:** Characteristics of study population

**Age**	**Number**	**Percent %**
18-30	94	34.6
31-43	155	57.1
44-58	22	8.1

**Years of Work**		
1–10	182	67.1
11–21	77	28.4
22–35	12	4.4

**Clinical Setting**		
Emergency Department	33	12.2
Internal Department	44	16.2
Surgery Department	46	16.9
Gynecology Department	10	3.7
Psychology Department	24	8.9
Operating Room	32	11.8
Pediatrics Department	17	16.3
Other Department	65	24

**Marital Status**		
Single	47	17.3
Married	206	76
Divorced/Widow/Separate (Reference)	18	6.7

79 (29.1%) participants including 31 male and 48 female answered yes to this question “Have you experienced physically aggressive behavior in the last twelve months?”. There were significant association between sex and answer of this question (p=0.035). In 35 cases (44.3%) the patients were the source of physical violence and in 44 cases (55.6%) the members of patients’ family were the source (Table 2). Unfortunately, threatening the nurses with physical harm was frequent behavior. Even if the rates were small, it is sad to confront that some of these threats are real.

**Table 2:** Source of violence against the nurses in this series

Identity of Perpetrator	Verbal Abuse (N=260)	Physical Violence (N=79)
Member(S) of Patient’s Family or Friends	139(53.4 %)	44(55.6 %)
Patient	79(30.3 %)	35(44.3 %)
Supervisor, Coworker, Doctor	42(16.1 %)	0

260 (95.9%) participants answered yes to this question “Have you experienced verbal aggression in the last twelve months?”. 185 of the victims were female and 75 were male. There was significant association between sex and answer of this question (p=0.013). In 79 cases (30.3%) the patients were the source of verbal violence and in 139 cases (53.4%) the members of patients’ family were the source and in 42 cases (16.1%) coworkers were the source (Table 2).

## 4. Discussion

In this study, we investigated the prevalence of verbal and physical violence against the nurses in different ward of the university hospitals. The main research questions in the present literature were: “Have you experienced physically aggressive behavior in the last twelve months?” and “Have you experienced verbal aggression in the last twelve months?”

The main outcomes of this survey demonstrated that verbal violence was a prominent professional risk for health care professions. On average, 95.9% of the participants have experienced verbal abuse and 29.1% have experienced physical violence.

In a study, Shoghi et al. (2008) reported that Verbal abuse was noted by 87.4% of the nurses during a 6-month period, and physical violence by 27.6% during the same period. The majority of nurses reported that abuse was followed by either inaction or by actions which failed to satisfy the victim. Based on their results, men were exposed to more abuse than women. In contrast with our study, women exposed to more abuse.

Çelik et al. (2007) reported the prevalence of verbal and physical violence against nurses 91.1% and 33.0% respectively. Coworkers were indicated to be the most important source of verbally abusive behaviors while patients and patients’ families were the important sources of physical violence. But in the present survey member(s) of patient's family or friends were source of violence for both verbal and physical abuse.

Franz et al. (2010) studied the prevalence of violence which happened during one year in 123 nurses and health care employees in a cross-sectional survey. They reported during the previous twelve months 70.7% of the participants experienced physical and 89.4% verbal aggression. Abbas et al (2010) revealed 27.7% of nurses in their study experienced abuse of any kind, 69.5% verbal abuse; and 9.3% physical abuse. Males were more exposed to violence during the period of 12 months than females. They concluded Workplace violence against nurses is an important issue in Egypt. Likewise with our research majority of the nurses experienced violence during past 12 month.

All of these studies like our current survey revealed that the prevalence of verbal abuse is more common in hospitals than physical abuse. In this regard, some organizational procedures, like waiting for long time and difficult connections between professionals and patients/visitors, will make the patients and their family unstable and therefore increase the likelihood of violence (Hinson et al., 2003). Enough management procedures are required to improve such conditions (Chappell et al., 2006). Interactions that patient or visitors endure frustrating experiences also result in violence in general hospitals and health care centers. Examples involve the procedures which cause pain and/or anxiety and procedures that provoke the patient to feel as he or she does not receive treatment seriously (Winstanley, 2005). The patient's health state may cause violent incidents. For instance, patients with cognitive problems cannot find the conditions adequately. This indicates that some violence is more frequent in special wards.

## 5. Conclusion

As noted above, these conditions need specific interventions and the temporal resources of staff (Winstanley, 2004). There is a requirement to increase awareness about this significant problem among health care workers beside the general public.
